# Relationships between executive functions and sensory patterns among adults with specific learning disabilities as reflected in their daily functioning

**DOI:** 10.1371/journal.pone.0266385

**Published:** 2022-04-07

**Authors:** Kineret Sharfi, Sara Rosenblum, Sonya Meyer

**Affiliations:** 1 The Laboratory of Complex Human Activity and Participation, Department of Occupational Therapy, University of Haifa, Haifa, Israel; 2 Department of Occupational Therapy, Ariel University, Ariel, Israel; Radboud University, NETHERLANDS

## Abstract

Adults with specific learning disabilities (e.g., dyslexia, dysgraphia, and dyscalculia) reveal limitations in daily functioning in various life domains. Following previous evidence of deficient executive functions and unique sensory patterns in this population, this study examined how relationships between these two domains are expressed in daily functioning. Participants included 55 adults with specific learning disabilities and 55 controls matched by age, gender, socioeconomic status, and education. Participants completed a sociodemographic questionnaire, the Behavioral Rating Inventory of Executive Functions–adult version, and the Adolescent/Adult Sensory Profile. Results indicated significant relationships between executive functions (per the Behavioral Rating Inventory of Executive Functions–adult version) and sensory patterns (per the Adolescent/Adult Sensory Profile) as reflected in daily functioning. The low sensory registration pattern predicted 12% to 16% of the variance in the behavioral regulation index, metacognitive index, and general executive composite scores and was a significant predictor of specific executive function abilities. Results indicated that the difficulties of adults with specific learning disabilities in using executive function abilities efficiently might be tied to a high sensory threshold and passive self-regulation strategies. A deeper understanding of this population’s sensory–executive mechanisms may improve evaluation and intervention processes. This understanding can consequently increase executive abilities for improved daily functioning and life satisfaction.

## Introduction

Specific learning disabilities (SLD) are a group of neurodevelopmental disorders (e.g., dyslexia, dysgraphia, and dyscalculia) stemming from a biological origin that affects the brain’s ability to perceive or process verbal or nonverbal information efficiently and accurately [1]. Evidence supports the neurological origin of SLD, and there is a consensus that disturbed patterns of skill acquisition accompany SLD from the early development stages and throughout life [1–4]. The American Psychiatric Association’s *Diagnostic and Statistical Manual of Mental Disorders* [1] states that SLD interfere significantly with academic or occupational performance or with activities of daily living. However, due to the heterogenic nature of SLD, its definition has been debated worldwide for years [5–7].

Most studies about SLD have focused on children. Studies involving adults have focused mainly on their academic or work performance [8,9], whereas knowledge about possible underlying mechanisms of adults’ deficient performance abilities is still scarce [9]. In previous studies, we found deficient executive function (EF) abilities and unique sensory patterns among adults with SLD [10,11]. Thus, the aim of this study is to analyze the relationships between those two domains among this population.

Executive functions are higher-level cognitive functions associated with the frontal lobes that control lower-level processes and are involved in directed, effective activity [12–15]. They are responsible for generating adaptive responses congruent with daily performance situations in various life domains [12,16,17]. Poor EFs may affect most aspects of everyday life, including general quality of life, marital harmony, job success, and public safety [18].

The Behavior Rating Inventory of Executive Function-Adult Version (BRIEF-A) [19] is a self-report questionnaire reflecting EF abilities through daily performance scenarios. Evaluation of EF abilities using the BRIEF-A captures an ecological picture of an individual’s daily functional characteristics as they align with their EF abilities [20,21]. Thus, the BRIEF-A was implemented in both our previous and current studies.

Deficient EF abilities, including working memory, planning, task monitoring, and organization, have been found among adults with dyslexia [22]. In our previous study, significant correlations were found between decreased EF abilities, deficient daily organization in time ability, and decreased quality of life among adults with SLD [10]. Other results using the BRIEF-A revealed associations between EF deficiency and multiple aspects of functioning in everyday life among adults with ADHD [19,23].

Efficient performance in daily activities requires significant overlap and interaction between the brain areas that control the cognitive functions and the processing of sensory input [24,25]. Previous literature suggested that EFs are involved in modifying behavior and that they depend on new information from the sensory systems [12,26]. In this study, sensory patterns are discussed according to Dunn’s sensory processing framework, which suggests a relationship between a person’s self-regulation strategies and neurological thresholds [27,28]. *Self-regulation strategy* refers to the person’s strategic reaction to sensory stimuli on a passive-to-active continuum. A passive-response strategy means the person lets things happen and then reacts. In contrast, an active-response strategy means the individual actively does things to control the amount and type of input available to them [27,29].

The neurological threshold is the point at which there is enough sensory input to cause a nerve cell or neurological system to activate—that is, the person "notices it" [30]. Thresholds are situated on a continuum. Each person has a personal range of thresholds to notice and respond to sensory events in everyday life, and these thresholds may differ for each type of input [27,28,31].

The interaction of the threshold level and self-regulation strategy creates four sensory-processing patterns: (a) People with low registration (high threshold) and passive strategy do not notice sensory events, such as people’s appearances or dirt on their faces or hands, in daily life as readily as do others; (b) People with sensory sensitivity (low threshold) and passive strategy notice more sensory events than do others. These people are easily distracted; movements, sounds, smells, food textures, temperatures, and spices may make them feel uncomfortable more rapidly; (c) Sensation seekers (high threshold) and active strategy enjoy sensory experiences and find ways to enhance and extend those events, such as physical movements, mouth noises, or touching objects, in daily life; and (d) Sensory avoiders (low threshold) and active strategy find ways to limit sensory input throughout the day. They avoid distracting places where others may move, talk, or bump into them.

Each person may have different sensory patterns for different types of sensory stimuli [28,29]. Thus, classifying individuals by specific patterns is not possible. Importantly, however, it has been suggested that understanding the unique sensory patterns of populations with specific health conditions may have important practical implications for developing improved intervention programs [32]. Previous research had found impaired sensory processing among adults with SLD. For example, difficulties in processing auditory, visual, and phonological stimuli and tactile perception were reported among adults with dyslexia [33,34]. In addition, adults with SLD showed poorer sensitivity to information in speech input [35] and had higher levels of low registration, sensory sensitivity, and sensory-avoiding patterns than did controls [11].

The literature regarding the possible relationships between EF and sensory patterns among adults with SLD is scarce. However, it has been suggested that the brain’s capacity for gate response to irrelevant incoming sensory input may be a fundamental mechanism that prevents flooding higher brain structures with irrelevant information in adult patients with attention deficit hyperactivity disorder [36]. Lately, a relationship was reported between executive dysfunction and altered sensory processing with negative effects on the daily activity performance of older adults. The authors suggested that understanding the role of sensory processing, EFs, and the relationships between them in the elderly may contribute to prevention and intervention programs aiming to improve their daily function and quality of life [37]. Nevertheless, the underlying executive and sensory mechanisms in the daily functional limitations of adults with SLD are not fully understood. Therefore, examining the relationships between the sensory patterns of adults with SLD and their EFs as expressed in daily functioning may increase understanding of the underlying cognitive mechanisms related to their daily functional limitations.

This study is part of a larger study; therefore, some of the data were previously published [10,11]. The aims of this study were to examine relationships and predictive relationships between the unique sensory patterns and decreased EFs of adults with SLD as manifested through their daily functions, based on their self-reports, to gain further insight into their daily functioning limitations. Based on the results of previous studies [12,26,36], we hypothesized that the unique specific sensory patterns found among adults with SLD (i.e., the independent variable) might have predictive relationships with their decreased EFs (i.e., the dependent variable).

## Materials and methods

### Participants

The study used a convenience sample of 110 adults (55 with SLD and 55 controls) from the southern and central regions of Israel, recruited via social media. Upon contacting the researcher, potential participants were asked initial questions to confirm inclusion criteria: age 20 to 50 years, mother-tongue level of reading and writing Hebrew, vision and hearing intact or corrected with an aid, no motor or neurological disabilities, and generally healthy with no chronic diseases or significant injuries that affect performance. Participants in the study group presented proof of their SLD diagnoses by a formal professional. Participants were assigned to the control group if they answered "No" to the questions, "Has anyone ever told you that you may have a SLD?" and "Did you ever think you may have a SLD?".

The sample size was determined using G*Power3 [38], calculating a critical *t* value of 1.659 with 108 degrees of freedom and a sample power of 0.831 for 21 central constructs examined, with an effect size of 0.5 and alpha error probability of 0.05. The groups were matched for the sociodemographic variables of gender, age, education level, and socioeconomic status (α > 0.05).

### Instruments

#### Sociodemographic questionnaire

A 36-item self-report questionnaire was constructed for this study. It included information about the participants’ sociodemographic status, developmental background, high-school experiences, and employment history.

#### Behavioral rating inventory of executive functions, Hebrew version

The BRIEF-A, Hebrew version [39] is a self-report ecological questionnaire. It includes 75 items that examine participants’ behavioral manifestations of EFs related to their daily function (e.g., "I don’t plan ahead for future activities" or "I leave my room or home a mess") [39]. For each item, participants indicate on a 3-point scale (*never*, *sometimes*, and *often*) how frequently they behave as described. Nine clinical scale scores are calculated; higher scores indicate greater difficulties. *T* scores of 65 or higher are considered clinically significant [40].

Analysis of data from the BRIEF-A standardization sample yielded the two-factor solution used in this study: a behavioral regulation index (BRI), which includes the subscales of inhibition, shifting, emotional control, and self-monitoring and a metacognitive index (MI), which includes the subscales of task initiation, working memory, planning/organization, task monitoring, and organization of materials [41]. The clinical subscale and index scores were standardized to produce *t* scores according to age and gender norms. Finally, a global executive composite (GEC) score was calculated by adding the BRI and MI scores [39]. Initial results supported the Hebrew version’s internal consistency, structure, and discriminant validity among adults with attention deficit hyperactivity disorder [42]. In the current study, high internal consistency reliability was obtained for this version (mean α = .96).

#### Adolescent/Adult sensory profile, Hebrew version

The Adolescent/Adult Sensory Profile-Hebrew version (AASP) [43] is a standardized self-report questionnaire that includes 60 items related to sensory modulation and the subjects’ sensory processing. The questionnaire has norms for the general population aged 18 to 64 years and has good internal consistency psychometric properties [43,44]. Participants indicate how often they respond to each sensory event in the manner described on a 5-point Likert scale (1 = *almost never*; 5 = *almost always*). The 60 items are sorted into four subscales reflecting the different sensory processing patterns: low registration (e.g., "I do not seem to notice when someone touches my arm or back"), sensation seeking (e.g., "I seek out all kinds of movement activities"), sensory sensitivity (e.g., "I startle easily to unexpected or loud noises"), and sensation avoidance (e.g., "I stay away from noisy places"). In the current study, acceptable internal consistency reliability was obtained (α = .84).

### Procedure

The Ethics Committee for Human Subject Research, Faculty of Social Welfare and Health Sciences at Haifa University approved the study (confirmation no. 170/10). Participants signed written informed consent, and all personal identifiers were separated from the data. The data are available upon request via e-mail of the corresponding author. After obtaining signed written informed consent, the first author met participants individually in a quiet location. Each participant completed the sociodemographic questionnaire, followed by an extensive set of evaluations and questionnaires. Adults with SLD were offered to have the questions read aloud and a free professional advisory hour for their participation.

### Data analysis

Descriptive statistics were calculated for the sociodemographic characteristics, and Pearson correlations and a two-step hierarchical regression predictive model were conducted to examine relationships between EFs and sensory patterns. Stepwise regressions were conducted to predict EF capabilities (BRIEF-A general and subscale scores) by sensory pattern (AASP) scores. *Group* was entered as a possible predictor at the first step, followed by each sensory pattern to find their contribution to EF capabilities (BRIEF-A) scores beyond group membership.

## Results

### Sociodemographic characteristics

The participants with SLD (*n* = 55) comprised 34.5% men and 64.5% women aged 20 to 46 years (*M* = 29.58 years, *SD* = 6.4). The control group (*n* = 55) included 23.6% men and 76.4% women aged 23 to 47 years (*M* = 31.18 years, *SD* = 6.4). The socioeconomic status of most participants in both the study group (58.2%) and the control group (43.6%) was low; for the remaining participants, the average income was higher than average in the study regions. No significant between-group differences were found for age, gender, socioeconomic status, or education level. Within the SLD group, multiple (more than one) diagnoses were reported in 54 of the 55 participants: 33 (60%) participants were diagnosed with dyslexia, 41 participants (74.5%) with dysgraphia, and 22 participants (40%) with dyscalculia. In addition, 42 participants in the SLD group (76.4%) were diagnosed with ADHD as well as their SLD. For a more detailed description of the sample’s demographic characteristics, see Sharfi and Rosenblum [11].

### Descriptive characteristics of EFs (BRIEF-A) and sensory patterns (AASP) among adults with and without SLD

The means and standard deviations of the BRIEF-A subscale scores, indexes, and GEC scores for both study groups have previously been presented [10]. That study [10] further found significant between-group differences, with higher BRIEF-A scores indicating lower EF abilities in the SLD group than in the control group.

Both groups’ AASP scores also have been previously described [11]. That study’s [11] results revealed that although the sensory pattens of 80% to 81.8% of the control group were like the sensory patterns known in the general population, only 34.5% to 60% of the participants in the SLD group demonstrated such sensory processing levels. Specifically, 56.4% of participants in the SLD group had higher scores than most people in the low-registration sensory pattern, and 63.6% had higher scores than most people in the sensory-sensitivity pattern. The means and standard deviations of the AASP subscale scores in both groups revealed significant between-groups differences, with unique sensory patterns in the SLD group for the low-registration sensory pattern (*p* < 0.001), sensory-sensitivity pattern (*p* < 0.001), and sensory-avoidance pattern (*p* < 0.01). The lowest difference between the groups was found in their sensory-seeking pattern (*p* < 0.05).

### Relationships between EFs (BRIEF-A) and sensory patterns (AASP)

As presented in Table 1, this study found significant correlations between the general BRIEF-A scores: BRI, MI, and GEC and the low-registration sensory pattern in both groups. Significant correlations (*r* >. 3, *p* < .05) also were found between the general BRIEF-A scores and the sensory-sensitivity and sensory-avoidance scores among the SLD group.

**Table 1:** Correlations between executive function (BRIEF-A) General and Subscale Scores and Sensory-Pattern (AASP) scores in each group.

BRIEF-A	AASP subscale							
	Group with SLD (*n* = 55)				Control group (*n* = 55)			
	Low registration	Sensory sensitivity	Sensory avoidance	Sensory seeking	Low registration	Sensory sensitivity	Sensory avoidance	Sensory seeking
BRI	.48***	.33*	**.23**	**.13**	.31*	.27*	.15	-.06
MI	.62***	.45**	.37**	-.16	.36**	.03	.08	.19
GEC	.64***	.49***	.37**	-.06	.30*	.11	.28*	-.08
**Subscales**								
Inhibition	.36**	.29*	.22	.09	.34*	.19	.10	.10
Shifting	.49***	.54***	.53***	-.27*	.18	.05	.14	-.01
Emotional control	.40**	.26	.13	.24	.19	.40**	.14	-.22
Self-monitoring	.18	-.06	-.08	.23	.28*	-.01	.05	.08
Task initiation	.47***	.38**	.32*	-.15	.35**	.13	.08	.14
Working memory	.60***	.44**	.37**	-.11	.42**	.15	.14	.13
Planning/ organizing	.44***	.38**	.36**	-.13	.29*	.07	.13	.16
Task monitoring	.41**	.32*	.30*	-.11	.12	-.01	-.03	.10
Organization of materials	.52***	.47***	.25	-.18	.16	-.16	-.06	.14

AASP = Adolescent/Adult Sensory Profile; BRIEF-A = Behavioral Rating Inventory of Executive Functions; BRI = behavioral regulation index; MI = metacognitive index; GEC = global executive composite, SLD = specific learning disabilities.

*p ≤ 0.05

**p ≤ 0.01

***p ≤ 0.001.

The highest correlations (*r* = 54–64) among the SLD group were found between the low-registration sensory pattern and the BRIEF-A GEC and MI general scores and working memory and organization of materials subscale scores. Further, significant high correlations were found among this group between the BRIEF-A shifting subscale and the sensory-sensitivity (*r* = .54***) and sensory-avoidance (*r* = .53***) patterns.

Lower significant correlations were found in the control group between the low-registration sensory pattern and inhibition, task initiation, and working memory (*r* = .34*–.42**). Further, the emotional-control subscale score significantly correlated with the sensory-sensitivity pattern (*r* = .40**).

As presented in Table 1, no significant correlations were found between the sensory-seeking pattern and any BRIEF-A score in the control group. Only a low significant correlation (*r* = -.27*) was found in the research group, and that was with the shifting subscale.

After the correlation analysis results, we planned to insert *group* in the first step of the stepwise regression analysis followed by the other three sensory subscales (low registration, sensory sensitivity, and avoidance), excluding the sensory-seeking pattern for predicting the BRIEF-A EF scores. However, a preliminary analysis found significantly high correlation between sensory sensitivity and avoidance patterns (*r* = .69, *p* < .001), indicating collinearity. Thus, because higher correlations were found between sensory sensitivity and BRIEF-A scores, we kept sensory sensitivity but removed avoidance from the regression analysis. The sensory patterns that contributed to the explained variance are addressed in the following sections and related tables.

### Predicting EF (BRIEF-A) scores by sensory pattern (AASP) scores

#### Predicting BRIEF-A general scores

Table 2 presents results for predicting the general executive scores (BRI, MI, and GEC scores) above group membership. As shown in the table, the group accounted for 25% of the variance in BRI score prediction, and the low-registration sensory pattern accounted for 12% of the variance above group membership. *Group* also accounted for 41% of the MI score prediction, and low-registration sensory pattern added 16% to the prediction above group membership. When analyzing the prediction of the GEC score, the group accounted for 32% of the variability, and the low-registration sensory pattern added 16% to the GEC score prediction above group membership.

**Table 2:** Predicting each General Executive Function (BRIEF-A) Score by Sensory Patterns (AASP) subscale scores above group membership.

	BRIEF-A score								
	BRI			MI			GEC		
**Model 1**	*B*	*SE B*	β	*B*	*SE B*	β	*B*	*SE B*	β
Group	10.42	1.72	.50***	15.74	1.79	.64***	13.74	1.88	.57***
	Adj *R*^2^ = 0.25; *F*_(1,108)_ = 36.60***			Adj *R*^2^ = 0.41; *F*_(1,108)_ = 77.04***			Adj *R*^2^ = 0.32; *F*_(1,108)_ = 53.25***		
**Model 2**	*B*	*SE B*	β	*B*	*SE B*	β	*B*	*SE B*	β
Group	6.46	1.80	.31***	10.37	1.73	.42***	8.53	1.87	.36***
Low registration	5.58	1.20	.40***	7.58	1.16	.46***	7.35	1.26	.46***
	Adj *R*^2^ = 0.37; *F*_(2,107*)*_ = 32.52***			Adj *R*^2^ = 0.57; *F*_(2,107)_ = 74.62***			Adj *R*^2^ = 0.48; *F*_(2, 107)_ = 51.92***		

*N* = 110. AASP = Adolescent/Adult Sensory Profile; BRIEF-A = Behavioral Rating Inventory of Executive Functions; BRI = behavioral regulation index; GEC = global executive composite; MI = metacognitive index.

*p ≤ 0.05

**p ≤ 0.01

***p ≤ 0.001.

Please note: Although presented together in this table, separate stepwise regression analyses were conducted to predict each general score individually.

Following these results, regression analyses were conducted to examine the prediction of each BRI and MI subscale by the AASP sensory pattern scores.

Predicting each BRIEF-A BRI subscale score by sensory pattern (AASP) subscale score. Table 3 presents prediction models of each BRI subscale score previously found to be significantly correlated with the AASP sensory pattern score except for the self-monitoring subscale (i.e., it shows inhibition, emotional control, and shifting, as in Table 1). As shown in the table, the group accounted for 28% of the prediction variance in the inhibition score, and low registration added 8% of prediction above group membership. The group accounted for 8% of the variability of the emotional-control subscale, with low registration adding 8% to the prediction of this subscale score. As to the prediction of the shifting score, the group accounted for 18% of the variance. Sensory sensitivity added 10%, and low registration added another 4% to the prediction of shifting score. In all, both sensory patterns added 14% to the prediction of the shifting EF subscale, resulting in a third model (Model 3).

**Table 3:** Predicting each BRIEF-A BRI subscale score by AASP sensory pattern scores above group membership.

	BRIEF-A BRI Subscale								
	Inhibition			Emotional Control			Shifting		
**Model 1**	*B*	*SE B*	β	*B*	*SE B*	β	*B*	*SE B*	β
Group	11.800	1.790	.540***	6.382	1.970	.298	9.360	1.830	.440***
	Adj *R*^2^ = 0.28; *F*_(1,108)_ = 43.51***			Adj *R*^2^ = 0.08; *F*_(1,108)_ = 10.49*			Adj *R*^2^ = 0.18; *F*_(1,108)_ = 26.23***		
**Model 2**	*B*	*SE B*	β	*B*	*SE B*	β	*B*	*SE B*	β
Group	8.240	1.910	.370***	2.920	2.140	.140	5.410	1.980	.250**
Low registration	5.030	1.280	.340***	4.870	1.430	.340**			
Sensory sensitivity							5.050	1.260	.370***
	Adj *R*^2^ = 0.36; *F*_(2,107)_ = 32.34***			Adj *R*^2^ = 0.16; *F*_(2,107)_ = 11.55***			Adj *R*^2^ = 0.28; *F*_(2,107)_ = 22.97***		
**Model 3**							*B*	*SE B*	β
Group							4.25	1.99	.200*
Sensory sensitivity							3.34	1.43	.250*
Low registration							3.52	1.48	.250*
							Adj *R*^2^ = 0.32; *F*_(3,106)_ = 17.87***		

*N* = 110. AASP = Adolescent/Adult Sensory Profile; BRIEF-A = Behavioral Rating Inventory of Executive Functions; BRI = behavioral regulation index.

*p ≤ 0.05

**p ≤ 0.01

***p ≤ 0.001.

Please note: Although presented together in this table, separate regression analyses were conducted for each BRIEF-A BRI subscale score individually. The regression results of each subscale are presented from top down in each column.

#### Predicting BRIEF-A MI subscale scores by sensory patterns (AASP) subscale scores

Table 4 presents prediction models of each MI subscale score. Analysis to predict the BRIEF-A MI subscale scores from the AASP subscale scores above group membership revealed that the group accounted for 29% of the variance for task initiation, and low registration added 12% to the prediction above group membership. The group predicted 55% of the variance of working memory, and low registration added 13% to the variance prediction. The group predicted 26% of the planning/organization EF subscale, with low registration adding 11% of prediction. The group also accounted for 30% of the variance of task monitoring, with low registration adding 6% to this subscale prediction. The group further accounted for 14% of the variance of organization of materials subscale, while the low-registration pattern added 13% to the prediction.

**Table 4:** Predicting each specific BRIEF-A MI subscale score from AASP subscale scores above group membership.

	BRIEF-A MI subscale														
	Task initiation			Working memory			Planning/organization			Task monitoring			Organization of materials		
**Model 1**	*B*	*SE B*	β	*B*	*SE B*	β	*B*	*SE B*	β	*B*	*SE B*	β	*B*	*SE B*	β
Group	11.87	1.77	.54***	21.25	1.84	.74***	12.36	1.95	.52***	12.87	1.84	.56***	9.53	2.17	.39***
	Adj *R*^2^ = 0.29;*F*_(1,108)_ = 44.78***			Adj *R*^2^ = 0.55;*F*_(1,108)_ = 133.42***			Adj *R*^2^ = 0.26;*F*_*(*1,108)_ = 40.35***			Adj *R*^2^ = 0.30;*F*_(1,108)_ = 48.90***			Adj *R*^2^ = 0.14;*F*_(1,108)_ = 19.25***		
**Model 2**	*B*	*SE B*	β	*B*	*SE B*	β	*B*	*SE B*	β	*B*	*SE B*	β	*B*	*SE B*	β
Group	7.62	1.83	.35***	15.63	1.76	.55***	8.11	2.05	.34***	9.67	2.00	.42***	4.63	2.27	.19*
Low registration	6.00	1.23	.41***	7.93	1.18	.41***	6.00	1.37	.38***	4.52	1.34	.29**	6.90	1.52	.42***
	Adj *R*^2^ = 0.41; *F*_(2,107)_ = 39.02***			Adj *R*^2^ = 0.68; *F*_(2,107)_ = 116.27***			Adj *R*^2^ = 0.37; *F*_(2,107)_ = 33.07***			Adj *R*^2^ = 0.36; *F*_(2,107)_ = 32.48***			Adj *R*^2^ = 0.27; *F*_(2,107)_ = 21.61***		

*N* = 110. AASP = Sensory Profile-Adolescent/Adult version; BRIEF-A = Behavioral Rating Inventory of Executive Functions; MI = metacognitive index.

*p ≤ 0.05

**p ≤ 0.01

***p ≤ 0.001.

Please note: Although presented together in this table, separate regression analyses was conducted for each BRIEF-A MI subscale score individually.

Thus, the low-registration sensory pattern accounted for 6% to 13% of the variance in the prediction of these MI subscale scores. That pattern was a significant predictor of the MI task initiation scores, *F*(2,107) = 39.02, *p* < .001; working memory, *F*(2,107) = 116.27, *p* < .001; planning/organization, *F*(2,107) = 33.07, *p* < .001; task monitoring, *F*(2,107) = 32.48, *p* < .001; and organization of materials, *F*(2,107) = 21.61, *p* < .001, beyond the contribution of group membership (Table 4).

## Discussion

This study provides more insight into the relationships between executive functioning and sensory processing among adults with SLD compared to controls. The significant correlations between the GEC score and specific sensory patterns found in both groups—with higher correlations in the SLD group—align with previous findings of similar correlations among children with specific health conditions and those in the general population [45]. Previous results of this study that described more extreme sensory patterns in this population [11] may explain the finding of a higher level of significant correlations among the SLD group than among the control group. The significant correlations between specific EFs and the AASP low-registration sensory pattern can contribute to understanding a possible underlying mechanism of daily function in adults with SLD who present this sensory pattern of a high threshold. It has been suggested that difficulty in noticing relevant sensory input may limit the person’s ability to use specific EFs efficiently. For example, they may have limits in shifting attention to relevant cues, initiating task performance, using working memory, or planning and organizing steps and required materials towards achieving the goal [46].

Specific executive abilities also are correlated with the sensory sensitivity and sensory avoidance patterns, both of which relate to low sensory thresholds. For example, *shifting* correlated more with the sensory-sensitivity and sensory-avoidance patterns than with the low-registration sensory pattern. *Shifting* refers to the ability to modify a cognitive rule to guide behavioral choices and meet changing environmental demands. It involves moving between multiple tasks, operations, or mental sets [47,48]. This finding may suggest that adults with SLD who have low sensory thresholds, and therefore tend to notice sensory inputs including too many irrelevant stimuli, may experience a sensory overload that interferes with their ability to select relevant information for their EFs to use. In both cases (i.e., sensory patterns of the high and the low thresholds), the ability to efficiently perceive, select, and use sensory input may be negatively affected and lead to decreased EF abilities, as reflected through daily function and vice versa.

An interesting finding of this study is that no significant correlations were found between any EF score and the sensory-seeking pattern. Therefore, we hypothesized that being an active seeker of sensory input may be a compensating trait for having a high sensory threshold. In other words, perhaps using active seeking strategies to acquire relevant information enables efficient use of certain EF abilities that may assist people with SLD to obtain their daily goals.

Another result is that, despite previous findings of significantly deficient self-monitoring abilities in adults with SLD [10], no significant relationship was found in the current study between the participants’ executive self-monitoring abilities and their sensory-processing abilities. As such, it suggests that self-monitoring may be an intrinsic EF ability that does not depend on people’s sensory processing patterns. Interestingly, the BRIEF-A subscales that align with EF theories differentiate between the BRI self-monitoring subscale and the MI task-monitoring ability, which must depend on external information processing. In addition, the three BRIEF-A score categories differentiate between emotional and behavioral regulation. Further studies are needed to determine whether the type of information provided by the sensory systems may influence the person’s self-monitoring abilities in specific tasks with different sensory demands.

The prediction results add further insight into the relationships between EFs and sensory-processing abilities. In the group with SLD, the low-registration sensory pattern significantly predicted all general EF scores and all specific MI scores. It has been previously suggested that to identify the most relevant sensory input, the nervous system must integrate highly processed sensory data with intrinsic markers and decide which action to do (or not do) next [49]. Indeed, previous studies have linked various shared mechanisms, such as phonological awareness, working memory, auditory processing, visual attention, and language impairments, with reading and writing difficulties among this population [e.g., 50]. Such mechanisms need external environmental stimuli for efficient functioning. Accordingly, decreased EFs among students with SLD occurred as the students struggled to identify significant themes, initiate new tasks, or shift flexibly, especially in a complex and conceptually demanding academic curriculum that requires organization and synthesis of large amounts of information [51].

In this study, only the participants’ low-registration sensory pattern contributed to the prediction of their inhibition and emotional control. Possibly, people with low-registration sensory abilities may miss relevant information they need to consider before they respond or act. Missing such critical information may be tied with deficient inhibition and emotional control. A previous study found that a low-registration sensory pattern predicted sleep quality among adults with SLD [11]. Sleep quality plays a key role in learning and memory processes and relates to learning capacity and academic performance [e.g., 52,53]. Consequently, it is possible that constant daily experiences of missing information required for efficient functioning, as well as insufficient sleep, may have significant emotional implications for adults with SLD. Thus, these factors need to be evaluated in this population.

This study exhibits interesting associations between EFs and sensory processing patterns. Further studies are required to link sensory processing and EFs with daily functional deficiencies found among adults with SLD, such as in their organization in time abilities, which are meaningful for efficient function and were found to correlate significantly with their quality of life [10].

### Study limitations

Because of the volunteer nature of the participant sample, the gender ratio in this study sample does not represent that previously reported among the population with SLD [54]. Additionally, the participants’ EFs and sensory patterns were examined through self-reports about the participants’ daily behaviors. Future studies need to compare these findings with additional executive- and sensory-function measures and peer reports. Finally, due to the high heterogeneity in the SLD group, we suggest that more data is necessary to generalize from this sample to a larger population with SLD.

### Conclusions

The relationships found between EFs and sensory patterns of adults with SLD as reflected in their daily function may have theoretical and practical implications for rehabilitation processes in this population. At the theoretical level, these results may enhance the understanding of cognitive mechanisms that underlie the decreased daily function in various life domains previously reported among adults with SLD [9]. The current results suggest that some specific EF abilities, such as inhibition, emotional control, and self-monitoring, may be intrinsic and not depend on external sensory information for their efficient function. However, other specific EFs may depend on external sensory input and therefore may be decreased due to the unique sensory patterns previously reported among adults with SLD. Further, this study’s findings suggest that adults with SLD who have an active sensory-seeking pattern may use it to compensate for a high neurological threshold. At the clinical level, this study’s implications include the need to evaluate both the EFs and sensory patterns of adults with SLD to understand their daily functioning better. Finally, we suggest future studies examine whether individually tailored accommodations that include more intense task and environmental cues (e.g., repeating instructions, adding louder voices, enlarging visual cues, or adding a sense of touch) may improve input perception for adults with SLD and low-registration sensory patterns. If such accommodations improve sensory processing in this population, it may lead to increased EFs and better daily functioning among adults with SLD.
